# Supplementary material for: Diagnostic Performance of Positron Emission Tomography with Fibroblast-Activating Protein Inhibitors in Gastric Cancer: A Systematic Review and Meta-Analysis
Source: Int J Mol Sci. 2023 Jun 14;24(12):10136. doi: 10.3390/ijms241210136 (PMC10299436; doi:10.3390/ijms241210136)
Supplement: Supplementary file 1 [file ijms-24-10136-s001.zip › Supplementary table S2.pdf]

Excluded Studies	Reason for exclusion
Kratochwil, C.; Flechsig, P.; Lindner, T.; Abderrahim, L.; Altmann, A.; Mier, W.; Adeberg, S.; Rathke, H.; Röhrich, M.; Winter, H.; et al. 68Ga-FAPI PET/CT: Tracer Uptake in 28 Different Kinds of Cancer. *J Nucl Med* **2019**, *60*, 801–805, doi:10.2967/jnumed.119.227967.	Original Study not in the field of interest
Pang, Y.; Huang, H.; Fu, L.; Zhao, L.; Chen, H. 68Ga-FAPI PET/CT Detects Gastric Signet-Ring Cell Carcinoma in a Patient Previously Treated for Prostate Cancer. *Clin Nucl Med* **2020**, *45*, 632–635, doi:10.1097/RLU.0000000000003099.	Case report in the field of interest
Wang, G.; Jin, X.; Zhu, H.; Wang, S.; Ding, J.; Zhang, Y.; Yang, Z.; Wang, X. 68Ga-NOTA-FAPI-04 PET/CT in a Patient with Primary Gastric Diffuse Large B Cell Lymphoma: Comparisons with [18F] FDG PET/CT. *Eur J Nucl Med Mol Imaging* **2021**, *48*, 647–648, doi:10.1007/s00259-020-04946-0.	Case report not in the field of interest
Fan, C.; Guo, W.; Su, G.; Chen, B.; Chen, H. Widespread Metastatic Gastric Signet-Ring Cell Carcinoma Shown by 68Ga-FAPI PET/CT. *Clin Nucl Med* **2021**, *46*, e78–e79, doi:10.1097/RLU.0000000000003245.	Case report in the field of interest
Guo, W.; Chen, H. 68Ga FAPI PET/CT Imaging in Peritoneal Carcinomatosis. *Radiology* **2020**, *297*, 521, doi:10.1148/radiol.2020202469.	Case report not in the field of interest
Zhao, L.; Pang, Y.; Luo, Z.; Fu, K.; Yang, T.; Zhao, L.; Sun, L.; Wu, H.; Lin, Q.; Chen, H. Role of [68Ga]Ga-DOTA-FAPI-04 PET/CT in the Evaluation of Peritoneal Carcinomatosis and Comparison with [18F]-FDG PET/CT. *Eur J Nucl Med Mol Imaging* **2021**, *48*, 1944–1955, doi:10.1007/s00259-020-05146-6.	Original Study not in the field of interest
Lin, R.; Lin, Z.; Zhang, J.; Yao, S.; Miao, W. Increased 68Ga-FAPI-04 Uptake in Schmorl Node in a Patient With Gastric Cancer. *Clin Nucl Med* **2021**, *46*, 700–702, doi:10.1097/RLU.0000000000003623.	Case report in the field of interest
Song, Y.; Qin, C.; Liu, F.; Lan, X. Fibrous Dysplasia Mimicking Skeletal Metastasis on 68Ga-FAPI PET Imaging. *Clin Nucl Med* **2021**, *46*, 774–775, doi:10.1097/RLU.0000000000003671.	Case report not in the field of interest
Alan-Selçuk, N.; Ergen, S.; Demirci, E.; Ocak, M.; Kabasakal, L. [68Ga]DOTA-FAPI-04 PET/CT Imaging in a Case of a Signet Ring Cell Carcinoma of Stomach. *Eur J Nucl Med Mol Imaging* **2021**, *48*, 4523–4524, doi:10.1007/s00259-021-05213-6.	Case report in the field of interest
Pang, Y.; Zhao, L.; Meng, T.; Xu, W.; Lin, Q.; Wu, H.; Zhang, J.; Chen, X.; Sun, L.; Chen, H. PET Imaging of Fibroblast Activation Protein in Various Types of Cancer Using 68Ga-FAP-2286: Comparison with 18F-FDG and 68Ga-FAPI-46 in a Single-Center, Prospective Study. *J Nucl Med* **2023**, *64*, 386–394, doi:10.2967/jnumed.122.264544.	Original study in the field of interest, but enrolling patients with tumors other than gastric cancer
Şahin, E.; Elboğa, U.; Çelen, Y.Z.; Sever, Ö.N.; Çayırlı, Y.B.; Çimen, U. Comparison of 68Ga-DOTA-FAPI and 18FDG PET/CT Imaging Modalities in the Detection of Liver Metastases in Patients with Gastrointestinal System Cancer. *Eur J Radiol* **2021**, *142*, 109867, doi:10.1016/j.ejrad.2021.109867.	Original study in the field of interest, but enrolling patients with tumors other than gastric cancer
Chen, X.; Wei, M.; Wang, S.; Yang, Z.; Wang, X. Characterizing Concomitant Follicular Lymphoma and Gastric Carcinoma Using 68Ga-FAPI-04 and 18F-FDG PET/CT. *Clin Nucl Med* **2022**, *47*, 81–82, doi:10.1097/RLU.0000000000003781.	Case report in the field of interest
Huang, Y.; Cao, J.; Peng, D.; Liu, H.; Chen, Y. Bone Marrow Metastases From Gastric Adenocarcinoma on 68Ga-FAPI-04 PET/CT. *Clin Nucl Med* **2022**, *47*, 151–153, doi:10.1097/RLU.0000000000003839.	Case report in the field of interest
Peng, D.; He, J.; Liu, H.; Cao, J.; Wang, Y.; Chen, Y. FAPI PET/CT Research Progress in Digestive System Tumours. *Dig Liver Dis* **2022**, *54*, 164–169,	Review in the field of interest

doi:10.1016/j.dld.2021.07.011.	
Qiu, L.; Lan, L.; Liu, H.; Deng, J.; Chen, Y. 68Ga-FAPI PET/CT Detected Non-FDG-Avid Gastric Stromal Tumor. *Clin Nucl Med* **2022**, *47*, 226–227, doi:10.1097/RLU.0000000000003856.	Case report in the field of interest
Guo, Y.-H.; Yang, M.-F. Comparison of 18F-AlF-NOTA-FAPI and 18F-FDG Imaging in a Patient With Gastric Signet-Ring Cell Carcinoma. *Clin Nucl Med* **2021**, *46*, 929–930, doi:10.1097/RLU.0000000000003742.	Case report in the field of interest
Kou, Y.; Yao, Z.; Cheng, Z. Al18F-NOTA-FAPI-04 Outperforms 18F-FDG PET/CT in Identifying the Primary Lesion and Rare Metastases From Gastric Cancer. *Clin Nucl Med* **2021**, *46*, e570–e571, doi:10.1097/RLU.0000000000003727.	Case report in the field of interest
Mona, C.E.; Benz, M.R.; Hikmat, F.; Grogan, T.R.; Lueckerath, K.; Razmaria, A.; Riahi, R.; Slavik, R.; Girgis, M.D.; Carlucci, G.; et al. Correlation of 68Ga-FAPi-46 PET Biodistribution with FAP Expression by Immunohistochemistry in Patients with Solid Cancers: Interim Analysis of a Prospective Translational Exploratory Study. *J Nucl Med* **2022**, *63*, 1021–1026, doi:10.2967/jnumed.121.262426.	Original Study not in the field of interest
Gündoğan, C.; Güzel, Y.; Can, C.; Kaplan, İ.; Kömek, H. FAPI-04 Uptake in Healthy Tissues of Cancer Patients in 68Ga-FAPI-04 PET/CT Imaging. *Contrast Media Mol Imaging* **2021**, *2021*, 9750080, doi:10.1155/2021/9750080.	Original Study not in the field of interest
Erol Fenercioğlu, Ö.; Beyhan, E.; Arslan, E.; Çermik, T.F.; Ergül, N. Intense 68Ga-DOTA-FAPI-04 Uptake in Diffuse Sclerotic Skeletal Metastases of Gastric Cancer. *Clin Nucl Med* **2022**, *47*, e325–e326, doi:10.1097/RLU.0000000000004052.	Case report in the field of interest
Yang, T.; Ma, L.; Hou, H.; Gao, F.; Tao, W. FAPI PET/CT in the Diagnosis of Abdominal and Pelvic Tumors. *Front Oncol* **2021**, *11*, 797960, doi:10.3389/fonc.2021.797960.	Review in the field of interest
Çermik, T.F.; Ergül, N.; Yılmaz, B.; Mercanoğlu, G. Tumor Imaging With 68Ga-DOTA-FAPI-04 PET/CT: Comparison With 18F-FDG PET/CT in 22 Different Cancer Types. *Clin Nucl Med* **2022**, *47*, e333–e339, doi:10.1097/RLU.0000000000004073.	Original study not in the field of interest
Gilardi, L.; Airò Farulla, L.S.; Demirci, E.; Clerici, I.; Omodeo Salè, E.; Ceci, F. Imaging Cancer-Associated Fibroblasts (CAFs) with FAPi PET. *Biomedicines* **2022**, *10*, 523, doi:10.3390/biomedicines10030523.	Review not in the field of interest.
Af Burén, S.; Tran, T.A.; Klevebro, F.; Holstensson, M.; Axelsson, R. A 68Ga-FAPI-46 PET/CT Imaging Pitfall in Assessing Residual Gastric Cancer Early After Chemotherapy. *Clin Nucl Med* **2022**, *47*, 644–645, doi:10.1097/RLU.0000000000004143.	Case report in the field of interest
Fu, L.; Huang, S.; Wu, H.; Dong, Y.; Xie, F.; Wu, R.; Zhou, K.; Tang, G.; Zhou, W. Superiority of [68Ga]Ga-FAPI-04/[18F]FAPI-42 PET/CT to [18F]FDG PET/CT in Delineating the Primary Tumor and Peritoneal Metastasis in Initial Gastric Cancer. *Eur Radiol* **2022**, *32*, 6281–6290, doi:10.1007/s00330-022-08743-1.	Original study in the field of interest, retracted from publication
Wang, S.; Huang, Q.; Dong, K.; Qin, C.; Wen, G. Double Trap Escape: Omental Nodular Hyperplasia Misdiagnosed as Metastasis by 18F-FDG PET/CT and 68Ga-FAPI PET/MRI in a Patient With Gastric Adenocarcinoma. *Clin Nucl Med* **2022**, *47*, 551–552, doi:10.1097/RLU.0000000000004192.	Case report in the field of interest
Hotta, M.; Rieger, A.C.; Jafarvand, M.G.; Menon, N.; Farolfi, A.; Benz, M.R.; Calais, J. Non-Oncologic Incidental Uptake on FAPI PET/CT Imaging. *Br J Radiol* **2023**, *96*, 20220463, doi:10.1259/bjr.20220463.	Review not in the field of interest

Fendler, W.P.; Pabst, K.M.; Kessler, L.; Fragoso Costa, P.; Ferdinandus, J.; Weber, M.; Lippert, M.; Lueckerath, K.; Umutlu, L.; Kostbade, K.; et al. Safety and Efficacy of 90Y-FAPI-46 Radioligand Therapy in Patients with Advanced Sarcoma and Other Cancer Entities. *Clin Cancer Res* **2022**, *28*, 4346–4353, doi:10.1158/1078-0432.CCR-22-1432.	Original article not in the field of interest
Tatar, G.; Beyhan, E.; Erol Fenercioğlu, Ö.; Arslan, E.; Çermik, T.F. Comparison of 18F-FDG and 68Ga-FAPI PET/CT in Gastric Kaposi Sarcoma. *Clin Nucl Med* **2022**, *47*, e596–e599, doi:10.1097/RLU.0000000000004172.	Case report not in the field of interest
Qin, C.; Song, Y.; Gai, Y.; Ruan, W.; Liu, Q.; Liu, F.; Zheng, D.; Zhang, P.; Liu, H.; Zhang, T.; et al. Gallium-68-Labeled Fibroblast Activation Protein Inhibitor PET in Gastrointestinal Cancer: Insights into Diagnosis and Management. *Eur J Nucl Med Mol Imaging* **2022**, *49*, 4228–4240, doi:10.1007/s00259-022-05847-0.	Original study in the field of interest, but enrolling patients with tumors other than gastric cancer
Veldhuijzen van Zanten, S.E.M.; Pieterman, K.J.; Wijnhoven, B.P.L.; Pruis, I.J.; Groot Koerkamp, B.; van Driel, L.M.J.W.; Verburg, F.A.; Thomeer, M.G.J. FAPI PET versus FDG PET, CT or MRI for Staging Pancreatic-, Gastric- and Cholangiocarcinoma: Systematic Review and Head-to-Head Comparisons of Diagnostic Performances. *Diagnostics (Basel)* **2022**, *12*, 1958, doi:10.3390/diagnostics12081958.	Review in the field of interest
Deng, M.; Cai, L. Detection of Occult Gastric Adenocarcinoma by 68Ga-FAPI-04 PET/CT. *AJR Am J Roentgenol* **2023**, *220*, 444, doi:10.2214/AJR.22.28325.	Case report in the field of interest
Pang, Y.; Zhao, L.; Meng, T.; Xu, W.; Lin, Q.; Wu, H.; Zhang, J.; Chen, X.; Sun, L.; Chen, H. PET Imaging of Fibroblast Activation Protein in Various Types of Cancer Using 68Ga-FAP-2286: Comparison with 18F-FDG and 68Ga-FAPI-46 in a Single-Center, Prospective Study. *J Nucl Med* **2023**, *64*, 386–394, doi:10.2967/jnumed.122.264544.	Original study not in the field of interest
Beyhan, E.; Erol Fenercioğlu, Ö.; Yarikkaya, E.; Çermik, T.F.; Ergül, N. Chronic Gastritis Mimicking Malignancy on 68 Ga-FAPI-04 PET/CT. *Clin Nucl Med* **2023**, *48*, e37–e39, doi:10.1097/RLU.0000000000004439.	Case report in the field of interest
Huang, D.; Wu, J.; Zhong, H.; Li, Y.; Han, Y.; He, Y.; Chen, Y.; Lin, S.; Pang, H. [68Ga]Ga-FAPI PET for the Evaluation of Digestive System Tumors: Systematic Review and Meta-Analysis. *Eur J Nucl Med Mol Imaging* **2023**, *50*, 908–920, doi:10.1007/s00259-022-06021-2.	Review in the field of interest
Li, T.; Jiang, X.; Zhang, Z.; Chen, X.; Wang, J.; Zhao, X.; Zhang, J. Case Report: 68Ga-FAPI PET/CT, a More Advantageous Detection Mean of Gastric, Peritoneal, and Ovarian Metastases from Breast Cancer. *Front Oncol* **2022**, *12*, 1013066, doi:10.3389/fonc.2022.1013066.	Case report not in the field of interest
Zhang, Z.; Jiang, H.; Zhang, L.; Cheng, C.; Zuo, C. 68Ga-FAPI-04 PET/CT Versus 18F-FDG PET/CT in the Detection of Malignant Gastrointestinal Stromal Tumor. *Clin Nucl Med* **2023**, *48*, 61–63, doi:10.1097/RLU.0000000000004364.	Case report in the field of interest
Fu, L.; Huang, S.; Wu, H.; Dong, Y.; Xie, F.; Wu, R.; Zhou, K.; Tang, G.; Zhou, W. Retraction Note: Superiority of [68Ga]Ga-FAPI-04/[18F]FAPI-42 PET/CT to [18F]FDG PET/CT in Delineating the Primary Tumor and Peritoneal Metastasis in Initial Gastric Cancer. *Eur Radiol* **2023**, doi:10.1007/s00330-022-09386-y.	Retraction note
Wang, Y.; Luo, W.; Li, Y. [68Ga]Ga-FAPI-04 PET MRI/CT in the Evaluation of Gastric Carcinomas Compared with [18F]-FDG PET MRI/CT: A Meta-Analysis. *Eur J Med Res* **2023**, *28*, 34, doi:10.1186/s40001-023-00997-9.	Review in the field of interest

Shu, Q.; Wang, Y.; Deng, M.; Chen, X.; Liu, M.; Cai, L. Benign Lesions with 68Ga-FAPI Uptake: A Retrospective Study. *Br J Radiol* **2023**, *96*, 20220994, doi:10.1259/bjr.20220994.	Original study not in the field of interest
Chen, R.; Yang, X.; Ng, Y.L.; Yu, X.; Huo, Y.; Xiao, X.; Zhang, C.; Chen, Y.; Zheng, C.; Li, L.; et al. First Total-Body Kinetic Modeling and Parametric Imaging of Dynamic 68Ga-FAPI-04 PET in Pancreatic and Gastric Cancer. *J Nucl Med* **2023**, jnumed.122.264988, doi:10.2967/jnumed.122.264988.	Original study in the field of interest, but enrolling patients with tumors other than gastric cancer
